# Intraductal Delivery of Adenoviruses Targets Pancreatic Tumors in Transgenic Ela-myc Mice and Orthotopic Xenografts

**DOI:** 10.18632/oncotarget.795

**Published:** 2013-01-12

**Authors:** Anabel José, Luciano Sobrevals, Juan Miguel Camacho-Sánchez, Meritxell Huch, Núria Andreu, Eduard Ayuso, Pilar Navarro, Ramon Alemany, Cristina Fillat

**Affiliations:** ^1^ Institut d'Investigacions Biomèdiques August Pi i Sunyer (IDIBAPS), Barcelona; ^2^ Centro de Investigación Biomèdica en Red de Enfermedades Raras (CIBERER), Barcelona; ^3^ Laboratori de Recerca Traslacional IDIBELL-Institut Catal&agrave; d'Oncologia, L'Hospitalet de Llobregat; ^4^ Centre de Biotecnologia Animal i Teràpia Gènica and Centro de Investigación Biomédica en Red de Diabetes y Enfermedades Metabólicas Asociadas CIBERDEM, Barcelona; ^5^ Institut de Recerca Hospital del Mar-IMIM, Barcelona, Spain

**Keywords:** Pancreatic cancer, adenovirus, orthotopic xenografts, transgenic mice, thymidine kinase

## Abstract

Gene-based anticancer therapies delivered by adenoviruses are limited by the poor viral distribution into the tumor. In the current work we have explored the feasibility of targeting pancreatic tumors through a loco-regional route.

We have taken advantage of the ductal network in the pancreas to retrogradelly inject adenoviruses through the common bile duct in two different mouse models of pancreatic carcinogenesis: The transgenic Ela-myc mice that develop mixed neoplasms displaying both acinar-like and duct-like neoplastic cells affecting the whole pancreas; and mice bearing PANC-1 and BxPC-3 orthotopic xenografts that constitute a model of localized human neoplastic tumors. We studied tumor targeting and the anticancer effects of newly thymidine kinase-engineered adenoviruses both in vitro and in vivo, and conducted comparative studies between intraductal or intravenous administration. Our data indicate that the intraductal delivery of adenovirus efficiently targets pancreatic tumors in the two mouse models. The in vivo application of AduPARTK^T^ plus ganciclovir (GCV) treatment induced tumor regression in Ela-myc mice. Moreover, the intraductal injection of ICOVIR15-TK^T^ oncolytic adenoviruses significantly improved mean survival of mice bearing PANC-1 and BxPC-3 pancreatic xenografts from 30 to 52 days and from 20 to 68 days respectively (p<0.0001) when combined with GCV. Of notice, both AduPARTK^T^ and ICOVIR15-TK^T^ antitumoral responses were stronger by ductal viral application than intravenously, in line with the 38-fold increase in pancreas transduction observed upon ductal administration.

In summary our data show that cytotoxic adenoviruses retrogradelly injected to the pancreas can be a feasible approach to treat localized pancreatic tumors.

## INTRODUCTION

Pancreatic ductal adenocarcinoma (PDAC) is the predominant form of pancreatic cancer and is one of the most aggressive and devastating human malignancies in developed countries. It is the fourth leading cause of cancer related deaths and has an extremely poor prognosis, with a median survival of 6 months and a 5-year survival of less than 5%. Pancreatic cancer is usually diagnosed at late stages and only 10-15% of patients present with operable disease. About 25% of unresectable PDACs are locally advanced and the rest are metastatic. The only potentially curative treatment is complete surgical resection of the tumor. Currently, the survival of non-resected patients is not fundamentally altered by any particular general therapy [1].

Gene therapy and virotherapy represent promising new therapeutic modalities for cancer. Among them, oncolytic adenoviruses are at the front line of anticancer agents. They are engineered to specifically target, replicate in and destroy cancer cells [2-4]. The possibility to arm the viruses with transgenes has resulted in improved antitumoral agents. However, the efficacy of these therapies is highly dependent on the capacity of adenoviruses to enter tumor cells and to distribute throughout the tumor. Commonly, they are applied *in vivo* through intratumoral injections or by systemic delivery. However, both intratumoral and intravenous administrations result in reduced spread of the viruses throughout the tumor limiting their therapeutic impact [5].

The exocrine pancreas is a secretory organ from which cells release digestive enzymes or ions through a complex network of ducts. Such network is well organized in a ductal tree that branches from the main duct to small interlobular ducts, generating intralobular and intercalated ducts [6]. In the present study we have taken advantage of such ductal network to address the feasibility to target pancreatic tumors by the retrograde delivery of adenoviruses into the common bile duct. This technique is similar to the endoscopic retrograde cholangiopancreatography, which has been established as a safe procedure in humans [7] and previous studies have shown that adenovirus and adeno-associated virus can be efficiently delivered to the mouse pancreas by this approach [8, 9]. We have analyzed adenoviral tumor targeting in the transgenic Ela-myc mouse model of pancreatic tumorogenesis and in pancreatic orthotopic xenografts. Ela-myc mice express oncogene c-myc under the elastase promoter and develop mixed acinar-ductal tumors in a ductal architecture that resembles PDAC [10, 11] which makes them an interesting model to evaluate the potential of the intraductal delivery route; however, the study of adenoviral therapies is limited to the use of recombinant adenovirus because mouse cells are poorly permissive to human adenovirus replication [12]. Thus, to evaluate the potential therapeutic value of oncolytic adenoviruses when applied intraductally, human orthotopic xenografts have been used as a model. Antitumoral effects have been evaluated by administering TK-engineered adenoviruses: the recombinant AduPARTK^T^ and the oncolytic adenovirus ICOVIR15-TK^T^. Our data shows that intraductal administration is an effective delivery route to target tumors with adenoviruses and ICOVIR15-TK^T^ is a potent oncolytic adenovirus that provides with antitumor efficacy when combined with GCV. Noticeable significantly enhanced anticancer effects were obtained, upon intraductal administration compared to systemic injection. We propose the intraductal delivery as a novel route to administer adenoviruses to pancreatic cancer patients.

## RESULTS

### Intraductal delivery of reporter adenoviruses targets pancreatic tumors in transgenic Ela-myc mice

**Figure 1:**
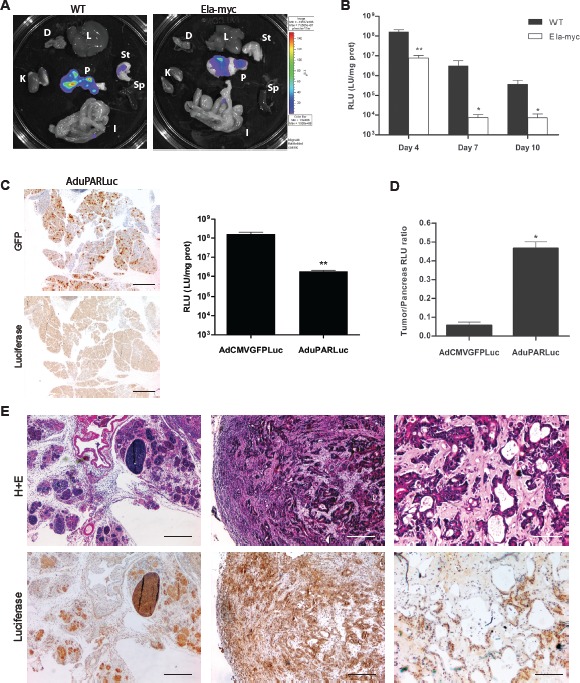
Expression of reporter adenoviruses intraductally administered into the common bile duct of wt and Ela-myc mice A, 5×10^10^ vp of AdCMVGFPLuc were intraductally administered to wt (n=7) or Ela-myc (n=9) mice and luciferase expression was measured four days later. (K: kidney, D: diafragm, L: liver, St: stomach, Sp: spleen, I: intestine and P: pancreas). B, 10^10^ vp of AdCMVGFPLuc were intraductally administered to wt (n=5) and Ela-myc mice (n=5), and luciferase expression was measured in pancreatic tissue extracts. C, D, 10^10^ vp of AdCMVGFPLuc or AduPARLuc were intraductally administered to wt (n=7 and 5, respectively) and Ela-myc mice (n=5); expression studies were performed 4 days later. C. Anti-GFP and anti-luciferase IHC in the pancreas of wt mice injected with AduPARLuc (left panel). Luciferase activity in the pancreas of wt mice (right panel). D. Cancer-specific index for each virus is expressed as the luciferase ratio tumor-pancreas (Ela-myc pancreas/ wt pancreas). E, Anti-luciferase IHC and H&E staining of pancreatic tissue sections from Ela-myc mice four days after i.d administration of AduPARLuc (10^10^ vp). Scale bars: 400 μm, 200 μm and 100 μm (left, middle and right panel, respectively). *p<0.05, ** p<0.01

AdCMVGFPLuc reporter adenovirus was injected into the common bile duct of 11-weeks-old transgenic Ela-myc and wild type (wt) mice. At this age all transgenic mice exhibited acinar neoplasms and 77% displayed a mixed acinar-ductal phenotype with tumor nodules ranging from 2-7 mm in diameter (Supplementary Fig. S1) [10]. Bioluminiscence imaging revealed luciferase expression restricted to the pancreas (Fig. 1A). Quantification of luciferase activity in tissue extracts showed higher activity in the pancreas of wt animals at all the time-points analyzed, that peaked at day 4 in both wt and Ela-myc mice (Fig. 1B). To enhance tumor selectivity AduPARLuc adenovirus was intraductally injected, previous data from our group has shown that AduPARLuc was highly active in tumor cells with reduced expression in normal tissue [13]. Taking advantage of the double cassette (CMVp/GFP and uPARp/Luc) in the AduPARluc virus, in the current study we show that AduPARLuc intraductally administered, broadly reaches the pancreas (strong GFP immunoreactivity) while uPAR promoter limits luciferase expression (weak luciferase signal) in normal pancreas (Fig. 1C, left panel). In line with these results, luciferase activity in the AduPARLuc injected pancreas was 2-log reduced when compared to AdCMVGFPLuc (Fig. 1C, right panel). Pancreatic tumor selectivity was confirmed in AduPARLuc injected animals showing a significantly higher (8,34-fold, p=0.016) cancer-specific index (established as a tumor to pancreas ratio) (Fig. 1D). This was in line with the observation that the uPAR gene was highly expressed in Ela-myc tumors (Supplementary Fig. S2). High intratumoral activity of uPAR controlled adenovirus was also evident from the analysis of anti-luciferase immunohistochemistry in Ela-myc tumors intraductally injected with AduPARLuc. Strong positive staining of acinar hyperplasic regions (Fig. 1E, left panel) and extensive areas of ductal-like tumor masses with dense stroma were observed (Fig. 1E, middle and right panels).

### Intraductally delivered AduPARTK^T^ adenovirus followed by GCV treatment triggers an antitumoral response in Ela-myc mice

**Figure 2:**
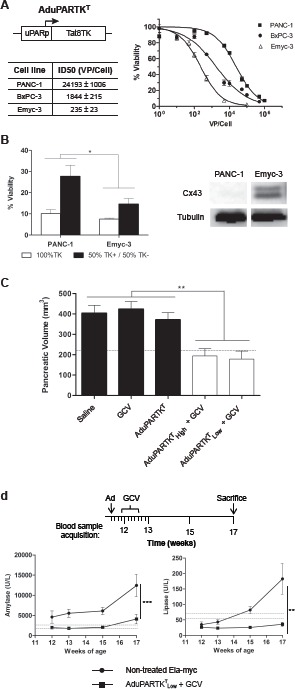
Antitumoral effects of AduPARTK^T^/GCV in pancreatic cancer cell lines and in Ela-myc mice A, Dose-response analysis of AduPARTK^T^/GCV treatment in PANC-1, BxPC-3 and Emyc-3 cells. B, Left: TK/GCV bystander effect assessed on 1:1 cocultures of TK+/TK- cells. Right: Connexin 43 expression analyses. C, Antitumoral effect of AduPARTK^T^/GCV therapy upon intraductal administration of the virus to Ela-myc mice followed by 6 doses of GCV. Pancreatic volume at six weeks after virus administration is plotted as indicator of antitumor effect. Dashed line corresponds to the pancreatic volume of wt mice (*n*=8). D, Analysis of amylase and lipase serum levels in non-treated (*n*=5) and treated Ela-myc mice (*n*=5). Dashed lines indicate reference values from untreated wt mice (*n*=6). * p<0.05, ** p<0.01, *** p<0.001

To evaluate the potential of adenoviral-based therapies to induce antitumoral effects upon application of this newly proposed route of virus administration, first we generated recombinant adenovirus expressing a modified form of the TK gene (TK^T^) under the control of the uPAR promoter (AduPARTK^T^). To examine the cytotoxic effects of the AduPARTK^T^/GCV suicide gene therapy, we first established Emyc cell lines from Ela-myc pancreatic tumors. These cell lines expressed ductal markers as well as the uPAR gene and were susceptible to adenoviral transduction (Supplementary Fig. S3). Cytotoxic evaluation of AduPARTK^T^/GCV was performed in Emyc cells and compared to BxPC-3 and PANC-1 human pancreatic cancer cells. To this end cells were transduced with increasing doses of AduPARTK^T^ and cell viability was analyzed after 3 days in culture in the presence of the prodrug GCV. MTT assays showed that the AduPARTK^T^/GCV treatment was able to induce cytotoxicity in Emyc cells to a larger extent than in PANC-1 or BxPC-3 cells, as evidenced by a shift to the left in the dose-response curve and by displaying the lowest ID_50_ value, indicating higher sensitivity to the treatment (Fig. 2A). To further analyze the cytotoxicity of the AduPARTK^T^/GCV system in Emyc cells, we evaluated for the presence of a bystander effect and compared it to that of PANC-1 cells, a cell line reported to have a moderate TK/GCV bystander effect [14]. Cells were transduced with AdTK at a viral dose corresponding to its ID_90_, and designated as TK^+^ positive cells. Cocultures of 50% TK^+^ and 50% TK^−^ cells were established at high confluence and treated with GCV for 3 days. Under these conditions the presence of a bystander effect would be detected when viability reached values below 55%. MTT analysis revealed that the survival of the Emyc-3 cocultures (15%) was significantly lower than that of PANC-1 (28%) (Fig. 2B, left panel). The enhanced effect in Emyc-3 cells could be, at least partially explained by the expression of connexin-43, a well-known mediator of the TK/GCV bystander effect (Fig. 2B, right panel) [14].

To analyze whether the cytotoxic effects of AduPARTK^T^/GCV would translate into an antitumor response, 11-weeks-old Ela-myc mice were randomly distributed into five groups. Mice received intraductal injections of PBS, AduPARTK^T^ (5×10^10^ vp) or AduPARTK^T^ (10^11^vp); three days later, when stated, a daily dose of GCV (100 mg/Kg) was administered for 6 consecutive days and six weeks after viral injection antitumor effects were determined. Given the fact that at 17-weeks of age the whole pancreas of Ela-myc mice displays carcinogenic lesions, the measure of pancreatic volume was used as an indicator of treatment effects. The three control groups that received PBS, GCV or AduPARTK^T^ alone showed large pancreatic volumes (1.8-fold over the pancreas of wild type mice). By contrast, animals treated with AduPARTK^T^ and GCV presented pancreatic volumes significantly smaller and similar to those of wild type animals. No differences were observed between the two viral doses (Fig. 2C). Of note, in a group of mice, tumor nodules observed at surgery completely regressed by treatment.

We reasoned that the presence of a large tumor in the pancreas could alter normal pancreatic function. In this line we assessed the levels of the pancreatic enzymes amylase and lipase in the serum of untreated Ela-myc mice. At 12-weeks of age Ela-myc mice already presented amylase levels above the reference range that dramatically increased by week 17. Lipase activity gradually increased over-time, reaching a 2.7-fold increase on the reference values by week 17 (Fig. 2D). Interestingly, AduPARTK^T^/GCV treatment resulted in normalization of the serum pancreatic enzymes (Fig. 2D).

These findings reveal that the intraductal delivery of AduPARTK^T^ followed by GCV reduces tumor progression and ameliorates pancreatic dysfunction in Ela-myc mice.

### Intraductal delivery of ICOVIR15-TK^T^ targets pancreatic tumors in orthotopic xenografts and increases mice survival

**Figure 3:**
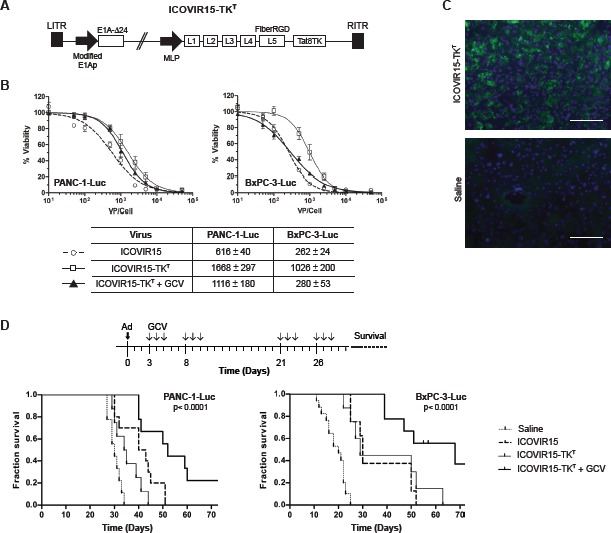
Antitumoral effects of ICOVIR15-TK^T^/GCV in pancreatic cancer cell lines and in human orthotopic tumors A, Schematic representation of ICOVIR15-TK^T^ virus. B, Dose-response analysis of ICOVIR15, ICOVIR15-TK^T^ and ICOVIR15-TK^T^ plus GCV in PANC-1-Luc and BxPC-3-Luc cells. C, Anti-E1A immunofluorescence of BxPC-3-Luc tumors, at 3 days after intraductal administration of ICOVIR15-TK^T^ (5×10^10^ vp). Scale bar: 100 μm. D, Antitumoral effect of ICOVIR15-TK^T^/GCV therapy upon intraductal administration of virus to mice bearing PANC-1-Luc and BxPC-3-Luc orthotopic pancreatic tumors. Kaplan-Meier analysis survival curves (log rank test, p<0.0001) are plotted.

To further validate the potential of intraductal delivery of cytotoxic adenoviruses as a therapeutic route for cancer treatment, we sought to study the potential antitumor effects of oncolytic viruses in pancreatic xenografts. We have previously shown that oncolytic adenoviruses expressing the TK gene when combined with GCV present enhanced antitumoral response and can be traced *in vivo* by PET imaging [15]. However, the limited genome encapsidation capacity of adenoviruses results in packaging problems when the TK gene is inserted. Interestingly a constrained genome size in an ICOVIR-15 backbone has been reported to allow for efficient transgene expression and oncolytic potency [16]. In this regard, we generated the ICOVIR15-TK^T^ by inserting the TK gene into the ICOVIR15 genome (Fig. 3A). Tumor selectivity of ICOVIR-15 was achieved by inserting eight E2F-binding sites and one Sp1-binding site in the E1A endogenous promoter. The TK gene was inserted downstream of the fiber gene under the control of the Major Late Promoter as previously reported [17]. The oncolytic potency of ICOVIR15-TK^T^ plus GCV was first studied *in vitro* and compared to ICOVIR15-TK^T^ without GCV and to the parental oncolytic virus ICOVIR-15. The cell killing effect was lower in PANC-1-Luc and BxPC-3-Luc cell cultures when infected with ICOVIR15-TK^T^ adenovirus, however the addition of GCV led to enhanced cytotoxicity. BxPC-3-Luc cells were more sensitive than PANC-1-Luc cells both to adenoviral cell killing and to TK/GCV cytotoxicity (Fig. 3B).

Next we assessed the capacity of oncolytic adenoviruses to target pancreatic tumors upon intraductal administration. To this end, 5×10^10^ vp of ICOVIR15-TK^T^ were intraductally injected into mice bearing BxPC-3-Luc tumors in the pancreas and E1A expression was evaluated in tumor sections. Strong E1A immunoreactivity was detected throughout the tumor (Fig. 3C). The antitumoral efficacy of ICOVIR15-TK^T^ was studied in the presence and the absence of GCV and compared to that of the parental oncolytic virus ICOVIR-15. Pancreatic xenograts from PANC-1-Luc and BxPC-3-Luc cells were treated with a single dose of saline, ICOVIR-15 or ICOVIR15-TK^T^ at 5×10^10^ vp/mouse administered intraductally. Mice injected with ICOVIR15-TK^T^ were split in two groups, one receiving intraperitoneal GCV (100 mg/Kg) at the time points indicated in the scheme, while the other group received saline solution (Fig. 3D). Treatment with ICOVIR-15 and ICOVIR15-TK^T^ improved survival in the two xenograft models. Interestingly an enhanced effect was observed with the combined treatment of ICOVIR15-TK^T^+GCV. Median overall survival increased from 30 days to 35 for ICOVIR15-TK^T^; to 42 for ICOVIR-15 and to 52 for ICOVIR15-TK^T^+GCV in the PANC-1-Luc model. Similarly, in the BxPC-3-Luc model overall survival increased from 20 days for untreated mice to 29 for ICOVIR15-TK^T^; 30 for ICOVIR-15 and to 68 for ICOVIR15-TK^T^+ GCV (Fig. 3D). Thus, the ICOVIR15-TK^T^ in combination with GCV resulted the most effective treatment in the two pancreatic xenograft models.

### Intraductal delivery of cytotoxic adenoviruses triggers a significantly higher antitumoral response than intravenous administration

**Figure 4:**
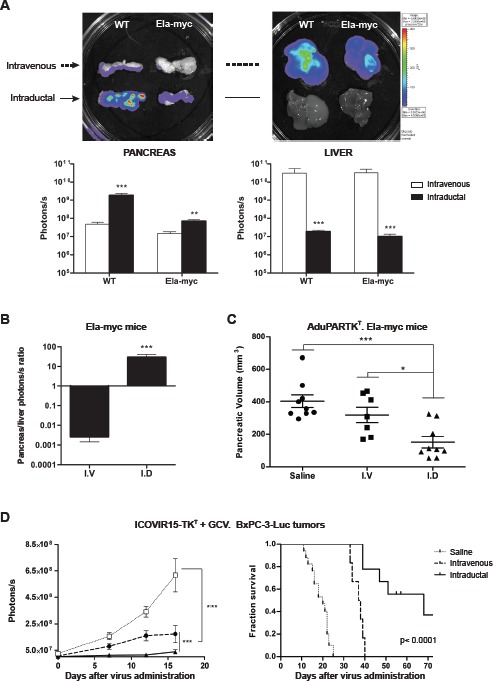
Comparative study of intraductal and intravenous delivery routes A, 5×10^10^ vp of AdCMVGFPLuc were intravenously or intraductally administered to wt (*n*=7 per group) and Ela-myc mice (*n*=7 per group). Luciferase expression was measured four days later. Top panel: Representative bioluminescent images. Bottom panel: Quantification of luciferase expression from captured bioluminescence images. B, Ratio pancreas/liver from Ela-myc mice. C, Antitumoral effect of AduPARTK^T^/GCV therapy upon intraductal or intravenous administration of AduPARTK^T^ (5×10^10^ vp) to Ela-myc mice. Pancreatic volume at six weeks after virus administration is plotted as indicator of antitumor effect. D, Antitumoral effect of ICOVIR15-TK^T^/GCV therapy upon intraductal or intravenous administration of ICOVIR15-TK^T^ (5×10^10^ vp) to mice bearing BxPC-3-Luc orthotopic pancreatic tumors. Tumor growth curves and Kaplan-Meier survival curves (log rank test, p<0.0001) are plotted. p<0.05, ** p<0.01, *** p<0.001.

Anticancer adenoviral based therapies are often applied through intravenous delivery. This route of administration is necessary to target disseminated cancer, however its hepatotoxic related effects encourage the use of alternative routes for the treatment of localized tumors. We sought to perform a comparative study between the intraductal (i.d) and intravenous (i.v) delivery of adenoviruses and assess tumor targeting efficiency and therapeutic response. First, 5x 10^10^ viral particles of AdCMVGFPLuc were injected into the tail vein or the common bile duct of wild type and Ela-myc mice. Four days later animals were sacrificed and bioluminescence was measured and quantified in the pancreas and the liver of injected animals. As shown in figure 4A following intravenous injection the strongest signal was detected in the liver at similar levels in both wild type and Ela-myc mice, whereas lower luciferase expression was detected in the pancreas. On the contrary, intraductal injection resulted in elevated luciferase activity in the pancreas and much lower values were detected in the liver (Fig. 4A). Noticeably, adenoviruses reached the pancreas both in wt and Ela-myc mice more efficiently upon intraductal delivery than following intravenous injection (38-fold and 5-fold respectively). Moreover, in Ela-myc mice the pancreas to liver ratio was significantly higher following intraductal delivery suggesting that adenoviruses reached more efficiently pancreatic tumors (Fig. 4B). Furthermore the enhanced tumor/liver ratio is an indication that through this route adenoviral-induced liver damage effects could be minimized.

To compare the antitumor efficacy of cytotoxic adenoviruses following intravenous or intraductal administration, AduPARTK^T^ were injected i.v. or i.d to Ela-myc mice and 3 days later they received GCV for six consecutive days. Six weeks after viral administration mice were sacrificed and the pancreatic volume was measured. Saline injected animals presented a large pancreas, indicative of tumor progression, whereas both i.v. and i.d injected mice showed reduced volume demonstrating antitumor efficacy. The major effect was observed in mice that received the virus intraductally (Fig. 4C).

To further determine the therapeutic potential of i.d. delivery, antitumor efficacy was also evaluated by applying 5x 10^10^ vp of oncolytic ICOVIR15-TK^T^ i.v. or i.d followed by GCV treatment in BxPC-3-Luc pancreatic xenografts, as scheduled in Fig. 3D. Tumor progression was analyzed by measuring luciferase expression. An exponential increase in luciferase activity was detected in the control group. By contrast, ICOVIR15-TK^T^ i.v delivered showed a slight increase on luciferase activity over time indicating limited tumor growth; interestingly the intraductal delivery of ICOVIR15-TK^T^ resulted in similar luciferase activity at all the time-points analyzed indicative of no tumor growth (Fig. 4D, left panel). Furthermore, ICOVIR15-TK^T^+GCV prolonged mouse survival after both intravenous and intraductal administration, although a remarkable increased survival was observed through intraductal delivery. The median survival in mice bearing BxPC3-Luc tumors was of 20 days for the mock group and of 38 days for the i.v. treated group and of 68 days for the i.d. treated group (Fig. 4D, right panel).

In summary, these results indicate that the intraductal delivery of adenoviruses is more successful than the intravenous delivery to target pancreatic tumors and induces stronger antitumoral effects.

## DISCUSSION

One of the major hurdles to meaningful treatment of pancreatic ductal adenocarcinoma by adenoviral-based therapies, is their relatively limited ability to spread throughout the tumor. In the present study we have explored the feasibility to achieve antitumoral effects by delivering adenoviruses by retrograde injection into the common bile duct with the aim to spread the virus into the tumor throughout the complex network of pancreatic ducts. We have tested this strategy in two different experimental models of pancreatic tumorogenesis, the transgenic Ela-myc mice that develop autochthonous tumors in the pancreas and a human cell transplantation model of orthotopic pancreatic xenografts. Our results show that in both models adenovirus, when delivered intraductally, are able to target the tumors generated in the pancreas as it can be visualized by the expression of luciferase in the pancreatic tumor nodules of transgenic mice and the immunodetection of E1A protein in the xenografted tumors. It has been proposed that tumor cells arising *in situ* in the native organ are distinct from cells engrafted into an immunocompromised mouse [18]. In this regard, tumors generated in the transgenic mice provide a more adequate tumor context for therapeutic evaluation. However, mouse cells are not permissive to human adenoviral replication thus, the study of antitumor effects of therapeutic adenoviruses in transgenic mice upon intraductal delivery was limited to the use of recombinant adenovirus expressing the suicide gene thymidine kinase (AduPARTK^T^) in combination with GCV. With this approach we observed a significant anticancer response at the two viral doses tested resulting in a pancreas size at week 6 after treatment similar to that of wild type animals, whereas control groups doubled the pancreatic mass. This good response was probably the result of a good sensitivity of the Ela-myc tumor cells to the TK/GCV cytotoxicity, as shown in the *in vitro* data, as well as to the spread of the virus into the tumor mass as a consequence of the intraductal adenoviral delivery. Furthermore, the strong activity of the uPAR promoter in the tumor tissue also represents a relevant contributing factor.

Oncolytic adenoviruses are promising agents for the treatment of cancer and the possibility to arm the virus with toxic transgenes further provides with enhanced potency. The potential of the intraductal delivery as a route for pancreatic cancer treatment with oncolytic adenoviruses was evaluated in orthotopic xenografts models. We have previously shown some of the advantages of the combined therapy of adenoviral replication and TK/GCV cytotoxicity: i) the improvement of the antitumoral response under a defined regimen of virus and GCV and ii) the possibility to monitor viral activity non-invasively by PET imaging based on TK expression [15, 17]. Here we report that GCV increases the cellular killing effect of the minimal RB responsive-based selective adenovirus armed with the TK gene (ICOVIR15-TK^T^), inducing significantly higher antitumor response than the potent oncolytic ICOVIR15 upon intraductal delivery in two different xenograft models of human pancreatic cancer BxPC-3-Luc and PANC-1-Luc. These data are in contrast to what is observed *in vitro* where ICOVIR15 is more active than ICOVIR15-TK^T^. The compromised activity of ICOVIR15-TK^T^ might be probably related to the limitations that adenoviruses have to efficiently package large genomes. ICOVIR15-TK^T^ has a 1.2Kb genome larger than ICOVIR15 what makes the virus less efficient in replication. The addition of GCV, which triggers the cell killing effect mediated by ganciclovir metabolites in ICOVIR15-TK^T^ +GCV treated cells fully compensates for this effect in BxPC-3-Luc cells, but only partially in the PANC-1-Luc model. The differences between cell lines may obey to their different sensitivity to cell death mediated by oncolysis or through TK/GCV cytotoxicity. However in vivo, viral replication does not seem to be a limiting factor, probably because there are many other tumor barriers that affect similarly to all the viruses. Interestingly, *in vivo* the treatment with ICOVIR15-TK^T^ +GCV produces a major antitumoral effect, probably because GCV toxic metabolites can spread to non-infected neighbouring tumoral or stromal cells increasing tumor cell destruction [19, 20]. The intraductal delivery of ICOVIR15-TK^T^ followed by GCV treatment induced a significantly higher antitumoral response than the same treatment when the virus was administered systemically. The improved anticancer response of intraductal compared to intravenous delivery was also observed in the AduPARTK^T^/GCV therapy in transgenic Ela-myc mice. This improved response was most probably related to an increased presence of adenoviral particles in the tumor upon intraductal delivery as suggested by the higher expression of luciferase in Ela-myc tumors after reporter virus injection. These results suggest that the spreading of the viral particles throughout the tumor mass is facilitated by the ductal network favoring tumor cell transduction. However it is important to note that the presence of the tumor stroma might impair a complete antitumor response. It is well known that the composition and structure of the extracellular matrix (ECM) can slow down the movement of molecules within the tumor [21]. In this line it could be speculated that the incorporation of an enzymatic agent that degrades ECM components could further facilitate viral tumor biodistribution and further extend survival. Recent reports have shown that the co-administration of hyaluronidase and an oncolytic adenovirus or the expression of hyaluronidase in an armed oncolytic virus improves the spread of the virus throughout the tumor [22, 23]. The benefits of hyaluronidase as an adjuvant regimen has also been shown to enhance gemcitabine anticancer effects [24, 25]. Therefore it would be worth to test the effects of co-treatment with hyaluronidase in adenoviral intraductal administrations.

The injection through the common bile duct or intraductal administration employed in the present study is an adaptation of the human ERCP technique. Although it is considered a safe technique, in around 10% of cases leads to a complication of acute pancreatitis which can be significantly reduced by administration of indomethacin [7, 26]. Transgenic Ela-myc mice already present signs of pancreatitis as shown by the elevated serum amylase and lipase levels. Although we can not discard that the intraductal delivery of adenovirus could induce additional pancreatitis at early stages, our data shows that since the initial phases of the treatment, AduPARTK^T^/GCV normalizes amylase and lipase serum levels. Another important aspect related to the safety of the delivery route is its minimal impact in other organs, as it is demonstrated by the higher ratio pancreas/liver achieved upon intraductal delivery when compared to intravenous administration in transgenic Ela-myc mice. This selectivity could minimize the liver-related toxicity associated to adenoviral systemic delivery [27]. Another major advantage of the intraductal delivery versus systemic administration is the potential to apply repeatable administrations. It is well known that readministration of an adenovirus of the same serotype is not indicated upon systemic delivery because it triggers the formation of neutralizing antibodies. Although we have not directly evaluated the feasibility of repeated administrations, Tominaga and collaborators showed with a very similar approach that readministration of adenoviral particles into the common bile duct of wild type mice resulted in efficient transgene expression without the need of any immunosuppressive strategies [28].

In summary, we have shown that retrograde intraductal adenoviral administration can be a feasible approach to efficiently and selectively target pancreatic tumors. Moreover the intraductal application of AduPARTK^T^ and ICOVIR-15TK^T^ in combination with GCV elicits significantly higher antitumor responses than the systemic delivery. These data highlight the potential of intraductal adenoviral delivery for the treatment of non-metastatic pancreatic neoplasms.

## METHODS

### Cell lines

HEK293 cells and the human pancreatic adenocarcinoma cell lines PANC-1, BxPC-3, were obtained from the American Type Cancer Collection (ATCC). PANC-1-Luc and BxPC-3-Luc were obtained and maintained as described previously [13, 29]. Emyc cells were established in our laboratory from pancreatic tumors of Ela-myc mice as described in Supplementary Methods. Cell lines were expanded as previously described [30]. Every 2 months, cells were plated from a frozen vial of the original batch but were not authenticated by the authors. Interspecies contamination was tested by PCR routinely.

### Adenovirus construction

Construction of AdCMVGFPLuc, AduPARLuc, AdTK and ICOVIR-15 have been previously described [3, 13, 16, 31, 32]. AdCMVGFPLuc and AduPARLuc express the enhanced GFP gene under the control of CMV promoter and the firefly luciferase gene under the control of CMV or uPAR promoter, respectively. AdTK adenovirus encodes the Herpes Simplex virus Thymidine Kinase (HSV-TK) under the control of a CMV promoter. In the present work we have generated the recombinant adenovirus AduPARTK^T^ and the oncolytic virus ICOVIR15-TK^T^. The AduPARTK^T^ recombinant adenovirus was generated by inserting the 1.2 kb sequence of the Tat8TK gene, a modified TK with enhanced cytotoxicity [33], into the NotI/XhoI sites of the pShuttle vector (Stratagene). Next, the SV40 polyA tail was cloned into the XhoI/XbaI sites of the previously generated plasmid, and the uPAR promoter (450 bp fragment) was inserted into the NotI site. Next, homologous recombination of the resulting pShuttle vector with the adenoviral genome was carried following a standard protocol. Viral particles were obtained and propagated in HEK293 cells. Oncolytic ICOVIR15-TK^T^ adenovirus was constructed by first generating the plasmid pICOVIR15-TK^T^. This plasmid was constructed by homologuos recombination in Saccharomyces cerevisiae [34] using SpeI-digested pICOVIR15 plasmid [16] as acceptor vector, and the SalI-PacI right end adenovirus genome fragment from plasmid pICOVIR5-TK-L [15] as donor insert. pICOIVR15-TK^T^ was cut with PacI and transfected in HEK293 cells to generate ICOVIR15-TK^T^, which was plaque isolated and propagated in A549 cells. All viruses were purified by standard cesium chloride banding and the physical particle concentration (vp/ml) was determined by optical density reading (OD_260_).

### Western blot analysis

Western blots were performed as previously described [14] and imaged on a LAS-3000 image analyzer (Fuji PhotoFilm Co.). Anti-Connexin43 (MAB3068, Chemicon International) and anti-α-tubulin (T9026, Sigma-Aldrich) were used as primary antibodies.

### In vitro cytotoxicity assays

PANC-1, PANC-1-Luc, BxPC-3, BxPC-3-Luc and Emyc-3 cells were transduced with AduPARTK^T^, ICOVIR15 or ICOVIR15-TK^T^ viruses, and cultured in the presence or absence of GCV (10 mg/ml, Cymevene) for 3 or 4 days. Cell viability was measured by an MTT colorimetric assay (Roche Molecular Biochemicals). ID_50_ values were estimated from dose-response curves by standard non-linear regression, using an adapted Hill Equation (Prism, version 5; GraphPad Software).

The TK/GCV bystander effect was measured by determining the viability of a mixed cell population composed of different percentages of TK+ (100 or 50%) and TK- (0 or 50%) cells, cultured for 3 days in the presence of GCV (10 μg/ml, Cymevene; Roche). The TK positive (TK+) cells were generated 24 h before establishing the cocultures by transduction with the recombinant adenovirus AdTK.

### Mouse models

Animal procedures met the guidelines of European Community Directive 86/609/EEC and were approved by the Local Ethical Committee. Transgenic Ela-myc and wt C57Bl6 mice of 11-17 weeks of age were used. Male athymic *nu/nu* mice (6-8 weeks old, Harlan Iberica) were used to generate orthotopic pancreatic tumors. Ela-myc mice were genotyped by multiplex PCR analysis as described in Supplementary Methods. Transgenic mice and wild type littermates were used in this study.

PANC-1-Luc and BxPC-3-Luc orthotopic tumors were generated by injecting 5×10^5^ cells into the pancreas of athymic nude mice, in a final volume of 50 μl, as previously described [29].

### Intraductal injection

Mice were anesthetized with a mixture of isofluorane and oxygen, and a mixture of buprenorphine (0.1 mg/Kg) and meloxicam (2 mg/Kg) was used as analgesic. A laparotomy of 2 cm was performed through a midline abdominal incision. Then the duodenum was exposed and the common bile duct was clamped close to the liver. A 30G needle was inserted into the duct from the duodenum through the ampulla of Vater, next the needle was clamped and 50 μl of virus were slowly injected (approximately 10 μl every 10 s). One minute later clamps were removed and the wound on the ampulla was closed with Histoacryl® (B.Braun). Abdominal muscle layer was closed with interrupted suture and the overlying skin was closed using Autoclips® (Stoelting Europe).

### Bioluminescence assay and quantification of luciferase expression

*In vivo* and *ex vivo* luciferase activity was visualized and quantified using an *in vivo* bioluminescent system (IVIS50; Xenogen; Caliper Life Sciences) and Living Image 2.20.1 Software overlay on Igor Pro4.06A software (Wavematrics) as previously described [35]. Luciferase transgene expression in tissue extracts was quantified using the reporter Luciferase Assay System (Promega) as previously described [13].

### Antitumoral efficacy

To evaluate the antitumoral capacity of AduPARTK^T^/GCV therapy, Ela-myc mice of 11 weeks of age were randomly divided in PBS (*n*=9), GCV (*n*=11), AduPARTK^T^ (*n*=9), AduPARTK^T^_High_+GCV (*n*=9) and AduPARTK^T^_Low_+GCV (*n*=7) groups and were intraductally injected with 5×10^10^ vp or 10^11^ vp of AduPARTK^T^ (Low and High, respectively). PBS and GCV groups received saline intraductally. AduPARTK^T^ group received 10^11^ vp of AduPARTK^T^. Three days later, GCV treatment (100 mg/Kg) was i.p administered for 6 days. Six weeks after virus administration, animals were sacrificed and the pancreas/tumor was removed (Fig. 2D). Pancreatic volume was measured and calculated according to the following formula: V(mm^3^) = *a* × *b* × *c* /2, where *a* = length, *b*= width, *c* = height of pancreas.

To evaluate the antitumoral efficacy of ICOVIR15-TK^T^, orthotopic PANC-1-Luc (*n*=8-10 per grup) and BxPC-3-Luc (*n*=8-9 per grup) pancreatic tumors were established. When tumors reached 10^6^-10^7^ photons/s measured by *in vivo* bioluminescence, which corresponds to approximately 100 mm^3^, animals were randomly divided into saline, ICOVIR-15, ICOVIR15-TK^T^ and ICOVIR15-TK^T^+GCV, and were intraductally or intravenously injected with 5×10^10^ vp of the corresponding adenovirus. Three days later GCV (100 mg/Kg) treatment was initiated, according to the protocol described in Fig.3D. Tumor growth was monitored by bioluminescence analysis. Survival studies were performed and animals were sacrificed according to ethical guidelines.

### Immunohistochemistry

Paraformaldehyde-fixed paraffin-embedded tissues and frozen tumor sections were prepared as previously described [29]. Five-micrometer paraffin-embedded sections were treated with 10 mM citrate buffer (pH 6.0) for antigen retrieval and incubated overnight at 4°C with anti-luciferase (L0159, Sigma) or with anti-GFP (A6455, Invitrogene) antibodies. Bound antibodies were detected with LSAB+ system-HRP (K0679, Dako). Tissue sections were counterstained with Harris's hematoxylin and mounted with EUKITT® (Sigma-Aldrich). Images were captured with a microscope (Leica DM6000 B) and digital camera (Leica DFC300 FX; Leica Microsystems) and processed with Leica Application Suite software. Immunofluorescence was performed in OCT-embedded sections by incubating with anti-adenovirus E1A (clone sc-430, Santa Cruz Biotechnology). Alexa Fluor 488 goat anti-rat antibody (Molecular Probes, Life Technologies™) was used as a secondary antibody. Nucleus were counterstained with 5 μg/ml bis-benzimide (Hoechst 33342; Sigma) and visualized under a fluorescent microscope (Observer/Z1; Zeiss). The fluorescent images were captured using a digital camera (AxioCamMRm; Zeiss).

### Pancreas toxicity study

Untreated 11-week-old Ela-myc mice or treated by intraductal injections of AduPARTK^T^ (5×10^10^ vp) followed by six doses of GCV (daily dose of 100 mg/Kg) were used for toxicology. Blood samples were collected at the indicated time-points. Serum amylase and lipase were determined on an Olympus AU400 Analyzer in the Clinical Biochemistry and Hematological Services of the Veterinary Faculty at the Autonomous University of Barcelona.

### Statistical analysis

Results are expressed as mean ± SEM of at least three independent experiments. Statistical differences were determined using Prism (version 5; GraphPad software) and were considered significant for P values less than 0.05. A Mann-Whitney nonparametric test was used for the statistical analysis (2-tailed) of *in vitro* studies. Regular two-way ANOVA was used to compare differences between the TK/GCV bystander effect in tumoral cell lines. Repeated-measures ANOVA was used to compare time-dependent differences among groups (pancreas toxicity study and ICOVIR15-TK tumor growth curves). Kruskal-Wallis test with Mann-Whitney U test for post hoc analyses was used to compare differences among AduPARTK^T^ treatment groups. Survival studies were performed to analyze time-to-event probability. The survival curves (Kaplan-Meier curves) obtained were compared for the different treatments. Animals that were alive at the end of the experiment were included as right censored information. A log-rank test was used to determine the statistical significance of the differences in time-to-event.

## Supplementary Figures


