# Supplementary material for: Intraductal Delivery of Adenoviruses Targets Pancreatic Tumors in Transgenic Ela-myc Mice and Orthotopic Xenografts
Source: Oncotarget. 2013 Jan 12;4(1):94–105. doi: 10.18632/oncotarget.795 (PMC3702210; doi:10.18632/oncotarget.795)
Supplement: Supplementary file 1 [file oncotarget-04-094-s001.pdf]

Intraductal Delivery of Adenoviruses Targets Pancreatic Tumors in Transgenic Ela-myc Mice and Orthotopic Xenografts

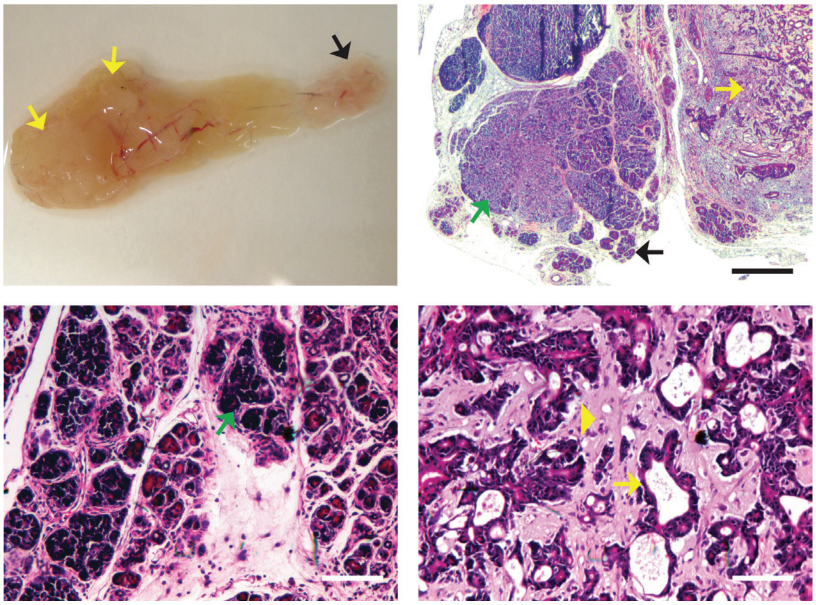

Supplementary Figure S1: Representative images and H&E staining of Ela-myc pancreas at 11 weeks of age. Black arrows indicate non-tumoral areas, yellow arrows indicate solid regions that show a ductal-like phenotype, yellow arrow head indicates dense stroma, green arrows indicate regions of acinar cell carcinoma. Scale bar: 400 µm (upper right panel), 100 µm (bottom panels).

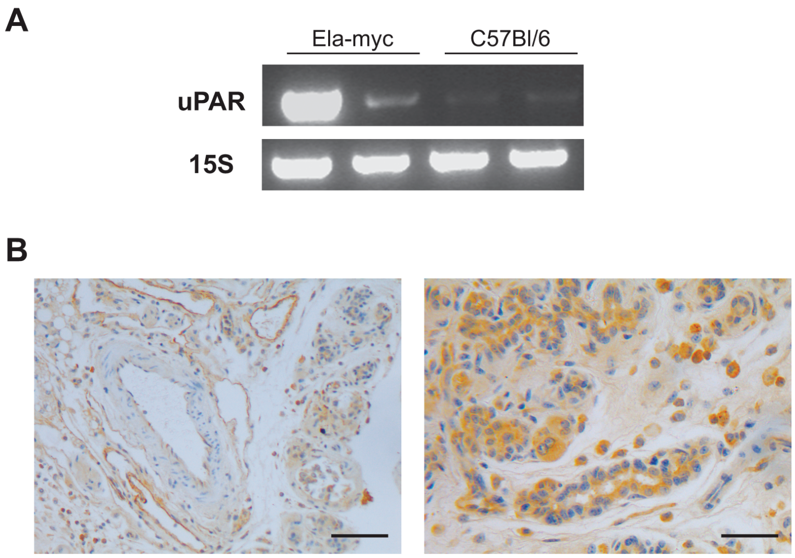

Supplementary Figure S2: uPAR expression in the pancreas of Ela-myc mice. A, RT-PCR of the Plaur gene (uPAR) and the control 15S in the pancreas of Ela-myc and wt mice. B, Representative images of anti-uPAR immunostaining in Ela-myc pancreas. Scale bar: 50 µm and 25 µm, left and right panel respectively.

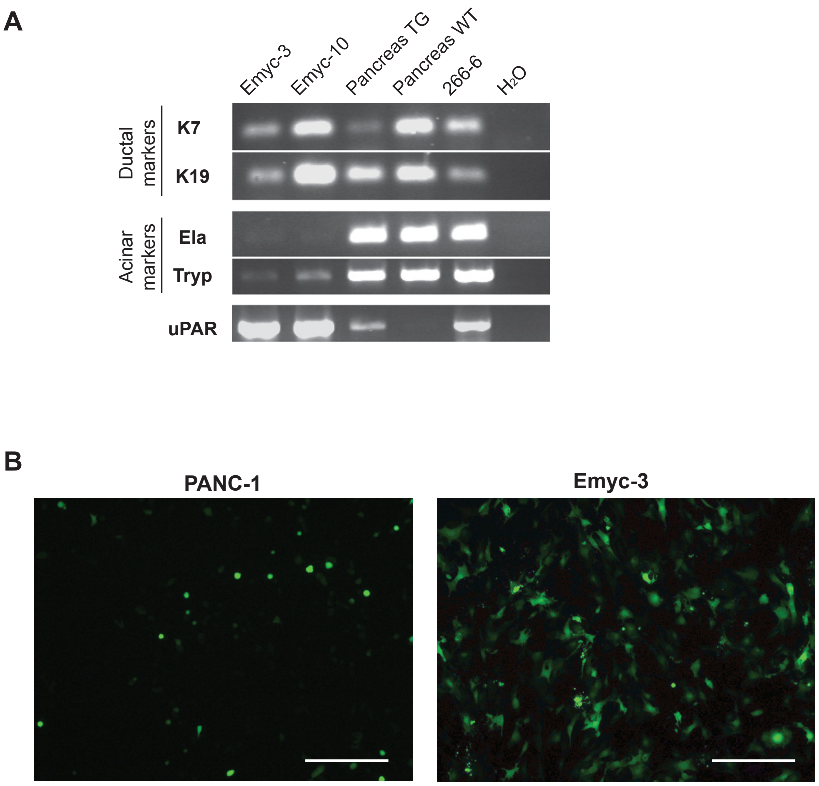

Supplementary Figure S3: Characterization of Emyc cell lines. A, Molecular characterization of Emyc-3 and Emyc-10 cell lines by RT-PCR analyses of ductal markers (cytokeratin 7 and cytokeratin 19), acinar markers (elastase and trypsin) and Plaur (uPAR) gene. Control lanes are pancreas of Ela-myc mice, pancreas of wt mice and the pancreatic acinar tumor cell line 266-6. B, Representative GFP fluorescent images of PANC-1 and Emyc-3 cells 48h after infection with 10.000 vp/cell of AdCMVGFPLuc. Scale bar: 400 µm.

SUPPLEMENTARY METHODS

Emyc cell lines generation. Emyc cells were established in our laboratory from pancreatic tumors from six independent Ela-myc mice of 3-6 months of age. Briefly, a small piece of the pancreatic tumor tissue was mechanically minced, placed into a culture dish and incubated in primary culture medium (DMEM supplemented with 20% FBS, 1x Non Essential Aminoacids, 20 ng/mL EGF, 200 U/ml penicillin, 200 µg/ml streptomycin and 0.25 µg/ml fungizone (Invitrogen)) at 37°C, in a humid atmosphere of 5% CO2. Cells were first passaged at 11-20 days by tripsinization and posterior filtration in a cell strainer (BD Falcon™). Around passage 20, a primary culture was considered an established cell line (4-5 months after initial plating).

Ela-myc genotyping: multiplex PCR analysis of c-Myc and Gdx (Myc Fw= 5' CACCGCCTACATCCTGTCCATTCAAGC 3', Myc Rv = 5' TTAGGACAAGGCTGGTGGGCACTG 3'; Gdx Fw = 5' GGCAGCTGATCTCCAAAGTCCTGG 3', Gdx Rv = 5' AACGTTCGATGTCATCCAGTGTTA 3'). PCR conditions were denaturation at 94°C for 20 sec, annealing a 63°C for 20 sec, and extension at 72C for 20 sec, for 35 cycles.
